# Association between lactate-to-albumin ratio and shortand long-term mortality in critically ill patients with ischemic stroke: A retrospective analysis of the MIMIC-IV database

**DOI:** 10.5937/jomb0-54979

**Published:** 2025-06-13

**Authors:** Sisi Qin, Jijie Xiao, Shiqi Yuan, Huitao Zhang, Yang Liu, Ningjun Li, Songjin He, Li Kou

**Affiliations:** 1 The Fifth Affiliated Hospital of Sun Yat-Sen University, Department of Critical Care Medicine, Zhuhai, Guangdong Province, China; 2 The Third Affiliated Hospital of Southern Medical University, Department of Radiology, Guangzhou, PR China; 3 Jinyang Hospital Affiliated to Guizhou Medical University, Neurology Ward 1, Guiyang City, Guizhou Province, China; 4 The Fifth Affiliated Hospital of Sun Yat-Sen University, Department of Neurology, Zhuhai, Guangdong Province, China

**Keywords:** lactate-to-albumin ratio, ischemic stroke, mortality, MIMIC-IV Database, odnos laktata i albumina, ishemijski moždani udar, mortalitet, MIMIC-IV baza podataka

## Abstract

**Background:**

Stroke is a major cause of disability and cognitive deficits, with ischemic stroke (IS) being the most prevalent type, especially in critically ill patients in intensive care units (ICUs). The lactate-to-albumin ratio (LAR) has emerged as a potential predictor of disease outcomes, but its association with shortand long-term mortality in critically ill IS patients is unclear.

**Methods:**

This study analyzed data from 894 critically ill IS patients from the MIMIC-IV database, categorized into LAR tertiles. Clinical endpoints included ICU, hospital, and 30and 90-day all-cause mortality. Survival differences were assessed using Kaplan-Meier analysis. Cox proportional-hazards regression models and restricted cubic spline (RCS) analysis evaluated the association between LAR and mortality outcomes. Subgroup analyses examined the modifying effects of clinical characteristics on LAR's predictive value.

**Results:**

The ICU, hospital, 30-, and 90-day mortality rates were 15.0%, 22.3%, 28.2%, and 36.1%, respectively. Higher LAR levels were associated with reduced survival times and increased mortality risks in all endpoints. Multivariable Cox models confirmed LAR as an independent predictor of 30and 90-day mortality. RCS analysis indicated a linear relationship between LAR and ICU mortality (P = 0.109), and a non-linear association with hospital (P = 0.005), 30-day (P < 0.001), and 90-day mortality (P < 0.001). Subgroup analyses highlighted significant interactions for respiratory failure and GCS.

**Conclusions:**

LAR is a robust predictor of shortand longterm mortality in critically ill IS patients, offering clinicians a valuable tool for risk stratification and decision-making.

## Abstract (sr)

**Uvod:**

Moždani udar je glavni uzrok invaliditeta i kognitivnih deficita, pri čemu je ishemijski moždani udar (IS) najčešći tip, posebno kod kritično bolesnih pacijenata u jedinicama intenzivne nege (ICU). Odnos laktata i albumina (LAR) se pojavio kao potencijalni prediktor ishoda bolesti, ali njegova povezanost sa kratkoročnim i dugoročnim mortalitetom kod kritično bolesnih pacijenata sa IS nije jasna.

**Metode:**

Ova studija je analizirala podatke od 894 kritično bolesna pacijenata sa IS iz baze podataka MIMIC-IV, kategorisane u LAR tertile. Kliničke krajnje tačke uključivale su intenzivnu negu, bolničku smrtnost i smrtnost od svih uzroka tokom 30 i 90 dana. Razlike u preživljavanju su procenjene primenom Kaplan-Meier analize. Koksovi regresijski modeli proporcionalnih opasnosti i analiza ograničenog kubnog splaj na (RCS) procenili su povezanost između LAR-a i ishoda mortaliteta. Analize podgrupa ispitivale su modifikacione efekte kliničkih karakteristika na prediktivnu vrednost LAR-a.

**Rezultati:**

Intenzivna intenzivnost, bolnička stopa mortaliteta 30 i 90 dana iznosila je 15,0%, 22,3%, 28,2% i 36,1% respektivno. Viši nivoi LAR-a bili su povezani sa skraćenim vremenom preživljavanja i povećanim rizikom od smrtnosti u svim krajnjim tačkama. Multivarijabilni Cok modeli potvrdili su LAR kao nezavisni prediktor mortaliteta od 30 i 90 dana. RCS analiza je pokazala linearnu vezu između mortaliteta LAR i intenzivne nege (P = 0,109) i nelinearnu povezanost sa bolničkim (P = 0,005), 30-dnevnim (P < 0,001) i 90-dnevnim mortalitetom (P < 0,001). Analize podgrupa su istakle značajne interakcije za respiratornu insuficijenciju i GCS.

**Zaključak:**

LAR je snažan prediktor kratkoročne i dugotrajne smrtnosti kod kritično bolesnih pacijenata sa IS, nudeći kliničarima dragoceno sredstvo za stratifikaciju rizika i donošenje odluka.

## Introduction

Stroke is a leading cause of disability and cognitive deficits globally, responsible for 5.2% of all deaths [1]. Ischemic stroke (IS), resulting from cerebral artery occlusion, is a primary cause of chronic disability worldwide, representing the majority of strokes [2] [3]. Pathophysiological changes post-ischemic stroke include cellular excitotoxicity, oxidative stress [4], ion imbalance, neuroinflammation, and abnormal immune cell activation, leading to neuronal death [2]. Critical stroke is prevalent among intensive care unit patients [5], highlighting the need for early disease severity assessment and intervention planning to reduce mortality. Consequently, there is a demand for simple, rapid, and practical biomarkers to predict and guide early intervention and treatment in IS patients.

Lactate, an indicator of tissue perfusion and circulatory shock [6], is rapidly produced in critically ill conditions characterized by hypoperfusion and hypoxia. Moreover, lactate has been considered a strong predictor of subsequent organ dysfunction and mortality [7]. Tissue acidosis is a sensitive metabolic marker for cerebral ischemic injury progression, primarily driven by lactate accumulation, which exacerbates neuronal ischemic damage [8] [9]. Approximately 60% of lactate is metabolized by the liver, 30% by the kidneys, and the remainder by other organs [6]. However, due to the influence of liver and kidney function, the use of lactate alone as an evaluation indicator lacks stability.

Serum albumin, in physiological mechanisms, serves various roles as an extracellular antioxidant, buffer, immunomodulator, detoxifier, and transporter protein in plasma [10] [11]. The properties of albumin are altered during ischemic attacks associated with oxidative stress, reactive oxygen species production, and acidosis [12]. Low serum albumin levels are epidemiologically linked to incident ischemic heart disease, heart failure, atrial fibrillation, stroke, and venous thromboembolism [13], indicating its potential in indicating lactate metabolic dysfunction and disease prognosis. In patients with stroke, albumin levels are inversely associated with stroke severity, degree of disability, and functional outcomes [14] [15] [16]. However, serum albumin synthesis is influenced by various factors, including colloid osmotic pressure, malnutrition, inflammation, diabetes, and liver disease [13], limiting its predictive value in IS.

Multiple studies have demonstrated the predictive value of the lactate-to-albumin ratio (LAR) for disease risk and prognosis in conditions such as acute pancreatitis [17], traumatic brain injury [18], sepsis [19], acute myocardial infarction [20], and cardiac arrest [21]. Examining the inverse changes in lactate and albumin, driven by distinct mechanisms, may hold significant prognostic value in IS patients. Even some literature has explored the relationship between LAR and 28-day all-cause mortality in ischemic stroke patients without reperfusion therapy [22]. However, the association between LAR and short- and long-term mortality in critically ill IS patients remains unclear. Therefore, using the Medical Information Mart for Intensive Care (MIMIC) IV database, this study aimed to determine the association between lactate-to-albumin ratio and both short- and long-term mortality in critically ill IS patients.

## Materials and methods

### Data source

The data analyzed in this study were sourced exclusively from the MIMIC-IV(v2.0) database, a publicly accessible repository derived from the electronic health records of the Beth Israel Deaconess Medical Center (https://physionet.org/content/mimiciv/2.0/), spanning the years 2008 to 2019 [23]. The MIMICIV (v2.0) database can be accessed and downloaded from the PhysioNet online forum (https://physionet.org/). Data extraction was conducted by the first author of this study, Sisi Qin, who has successfully completed the Collaborative Institutional Training Initiative (CITI) course, including passing both the »Conflicts of Interest« and »Data or Specimens Only Research« exams (ID: 51305476). It is important to note that all patient information in this database was anonymized, and the need for ethical review and informed consent was waived.

### Selection of participant

**Figure 1:**
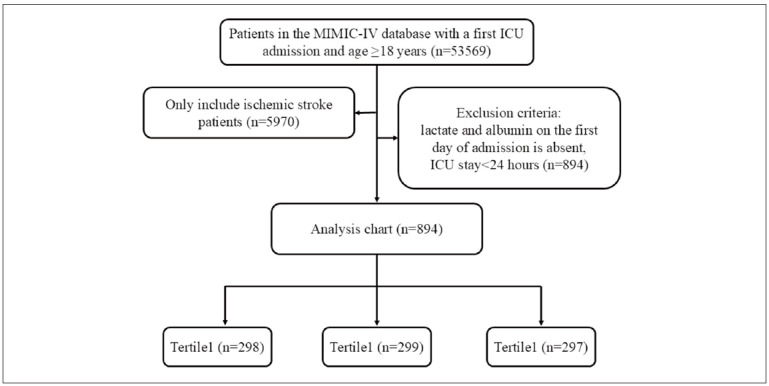
Flow of included patients through the trial.

All patients included in this study were selected from the MIMIC-IV(v2.0) database. The inclusion criteria were as follows: (1) identification of IS based on the ninth or tenth revision of the International Classification of Diseases code, and (2) adults ( 18 years) admitted to the ICU for the first time. The exclusion criteria were: (1) absence of lactate and albumin data on the first day of admission, and (2) ICU stay of less than 24 h. A total of 894 participants met the criteria and were included in the outcome cohort for the final analysis (Figure 1).

### Data extraction and outcomes

Raw data, encompassing baseline characteristics, comorbidities, vital signs, laboratory variables, and severity scores, was extracted from PostgresSQL (version 10.17) and Navicat Premium software using Structured Query Language (SQL) within the first day following ICU admission. Baseline characteristics, such as age and gender, were included. Comorbidities, such as hypertension, diabetes, heart failure, respiratory failure, renal disease, sepsis, and shock were identified using the ninth or tenth revision of the International Classification of Diseases code. Vital signs, including systolic blood pressure (SBP), diastolic blood pressure (DBP), and respiratory rate (RR), were recorded. Laboratory variables within the initial 24h after ICU admission comprised white blood cell count (WBC), hemoglobin (HGB), platelet counts (PLT), prothrombin time (PT), blood urea nitrogen (BUN), creatinine (Cr), sodium, and glucose levels. Treatment variables included antiplatelet therapy and mechanical ventilation. Severity scores upon admission were assessed using the Simplified Acute Physiology Score II (SAPS II) and Glasgow Coma Scale (GCS). The primary endpoints of this study were 30-day and 90-day mortality rates, while secondary endpoints included ICU and hospital mortality rates.

To mitigate potential bias, variables with a missing rate exceeding 20% were excluded. For variables with missing data below 20%, multiple imputation was performed using the »mice« package in R software. The imputation model that best aligned with the observed data was selected based on Akaike’s information criterion (AIC) and the Bayesian information criterion (BIC) [24].

### Statistical analysis

All participants were categorized into three groups based on the tertile of LAR for descriptive analysis (T1: LAR<0.411, T2: 0.411≤LAR< 0.715, and T3: LAR ≥0.715). The normality of continuous variables was assessed using the Kolmogorov-Smirnov test. Continuous variables are presented as mean ± SD for normally distributed data, median (IQR) for non-normally distributed data, and frequencies (%) for categorical variables. Baseline characteristics were compared using One-Way ANOVA for continuous variables, the Kruskal-Wallis test for non-normally distributed continuous variables, and the Chi-Squared Test for categorical variables. Inverse probability of treatment weighting (IPTW) was employed to minimize covariate differences and balance baseline characteristics across groups. Baseline differences among the three groups were analyzed using *p*-values (Table 1). Survival curves for different groups were generated using the Kaplan-Meier (KM) method and compared using the log-rank test. The association between LAR and short- and long-term all-cause mortality was assessed using the COX proportional-hazards regression model, with the first tertile as the reference group. Results were reported as hazard ratios (HRs) and 95% confidence intervals (CIs). Additionally, Restricted cubic spline (RCS) analysis was employed to explore the non-linear association between LAR and short- and long-term allcause mortality in critically ill patients with ischemic stroke. Subgroup analysis was conducted to assess the impact of LAR in different subgroups, including Age, Gender, Hypertension, Diabetes, Heart failure, Respiratory failure, Sepsis, Saps II, and GCS.

**Table 1:** Baseline characteristics between groups before and after IPTW.

Group	Level	Unmatched				IPTW			
		T1	T2	T3	*P*	T1	T2	T3	*P*
		298	299	297		304.92	291.79	301.79	
Age (mean (SD))		71.73<br>(14.47)	70.48<br>(14.87)	71.23<br>(15.53)	0.589	72.88<br>(13.73)	70.95<br>(14.65)	70.22<br>(15.80)	0.306
Gender (%)	F	138<br>(46.3)	128<br>(42.8)	141<br>(47.5)	0.492	153.4<br>(50.3)	136.1<br>(46.6)	126.7<br>(42.0)	0.382
	M	160<br>(53.7)	171<br>(57.2)	156<br>(52.5)		151.5<br>(49.7)	155.7<br>(53.4)	175.1<br>(58.0)	
Hypertension (%)	No	141<br>(47.3)	145<br>(48.5)	137<br>(46.1)	0.846	131.1<br>(43.0)	136.8<br>(46.9)	136.8<br>(45.3)	0.799
	Yes	157<br>(52.7)	154<br>(51.5)	160<br>(53.9)		173.8<br>(57.0)	155.0<br>(53.1)	165.0<br>(54.7)	
Diabetes (%)	No	187<br>(62.8)	179<br>(59.9)	164<br>(55.2)	0.169	179.5<br>(58.9)	177.2<br>(60.7)	180.1<br>(59.7)	0.945
	Yes	111<br>(37.2)	120<br>(40.1)	133<br>(44.8)		125.4<br>(41.1)	114.6<br>(39.3)	121.7<br>(40.3)	
Heart failure (%)	No	186<br>(62.4)	182<br>(60.9)	190<br>(64.0)	0.736	214.5<br>(70.3)	185.3<br>(63.5)	189.2<br>(62.7)	0.297
	Yes	112<bt>(37.6)	117<bt>(39.1)	107<bt>(36.0)		90.4<bt>(29.7)	106.4<bt>(36.5)	112.6<bt>(37.3)	
Respiratory failure (%)	No	185<br>(62.1)	150<br>(50.2)	137<br>(46.1)	<0.001	161.7<br>(53.0)	154.1<br>(52.8)	152.2<br>(50.4)	0.881
	Yes	113<br>(37.9)	149<br>(49.8)	160<br>(53.9)		143.2<br>(47.0)	137.7<br>(47.2)	149.6<br>(49.6)	
Renal disease (%)	No	283<br>(95.0)	288<br>(96.3)	287<br>(96.6)	0.546	293.9<br>(96.4)	279.6<br>(95.8)	280.3<br>(92.9)	0.352
	Yes	15<br>(5.0)	11<br>(3.7)	10<br>(3.4)		11.0<br>(3.6)	12.2<br>(4.2)	21.5<br>(7.1)	
Sepsis (%)	No	260<br>(87.2)	228<br>(76.3)	147<br>(49.5)	<0.001	225.1<br>(73.8)	209.8<br>(71.9)	216.8<br>(71.8)	0.893
	Yes	38<br>(12.8)	71<br>(23.7)	150<br>(50.5)		79.8<br>(26.2)	81.9<br>(28.1)	85.0<br>(28.2)	
Shock (%)	No	265<br>(88.9)	214<br>(71.6)	130<br>(43.8)	<0.001	216.8<br>(71.1)	205.0<br>(70.3)	206.1<br>(68.3)	0.847
	Yes	33<br>(11.1)	85<br>(28.4)	167<br>(56.2)		88.1<br>(28.9)	86.8<br>(29.7)	95.7<br>(31.7)	
Antiplatelet therapy (%)	No	65<br>(21.8)	87<br>(29.1)	126<br>(42.4)	<0.001	111.9<br>(36.7)	86.9<br>(29.8)	92.8<br>(30.8)	0.445
	Yes	233<br>(78.2)	212<br>(70.9)	171<br>(57.6)		193.0<br>(63.3)	204.9<br>(70.2)	209.0<br>(69.2)	
Mechanical ventilation (%)	No	143<br>(48.0)	127<br>(42.5)	101<br>(34.0)	0.002	129.6<br>(42.5)	122.6<br>(42.0)	130.9<br>(43.4)	0.97
	Yes	155<br>(52.0)	172<br>(57.5)	196<br>(66.0)		175.3<br>(57.5)	169.2<br>(58.0)	170.9<br>(56.6)	
SBP (mean (SD))		129.38<br>(25.72)	126.62<br>(27.01)	120.63<br>(26.36)	<0.001	123.61<br>(25.36)	126.14<br>(26.85)	124.91<br>(25.66)	0.664
DBP (mean (SD))		68.93<br>(19.39)	73.03<br>(20.20)	68.79<br>(20.43)	0.013	68.51<br>(20.52)	70.64<br>(19.11)	71.26<br>(19.35)	0.531
RR (mean (SD))		18.32<br>(5.74)	19.93<br>(6.12)	20.97<br>(6.62)	<0.001	19.19<br>(5.73)	19.50<br>(6.22)	19.76<br>(5.58)	0.697
WBC (mean (SD))		11.26<br>(6.07)	12.97<br>(6.89)	15.75<br>(9.15)	<0.001	12.64<br>(7.35)	12.89<br>(7.07)	12.99<br>(7.80)	0.933
HGB (mean (SD))		10.50<br>(2.35)	10.88<br>(2.39)	10.82<br>(2.46)	0.111	10.61<br>(2.28)	10.80<br>(2.43)	10.44<br>(2.50)	0.435
PLT (mean (SD))		200.72<br>(99.02)	203.94<br>(102.67)	197.43<br>(119.31)	0.761	197.77<br>(100.87)	201.66<br>(102.45)	202.38<br>(125.62)	0.925
PT (mean (SD))		14.81<br>(5.09)	15.81<br>(7.16)	21.06<br>(15.66)	<0.001	17.30<br>(9.53)	17.03<br>(11.41)	17.70<br>(10.28)	0.870
BUN (mean (SD))		26.59<br>(21.00)	30.68<br>(23.46)	37.49<br>(27.14)	<0.001	30.77<br>(21.32)	31.11<br>(24.11)	29.89<br>(23.91)	0.864
Cr (mean (SD))		1.52<br>(1.65)	1.54<br>(1.43)	1.84<br>(1.46)	0.017	1.53<br>(1.45)	1.65<br>(1.66)	1.61<br>(1.33)	0.723
Sodium (mean (SD))		138.33<br>(5.66)	139.04<br>(5.72)	140.30<br>(7.51)	0.001	140.03<br>(6.66)	139.14<br>(5.93)	138.94<br>(7.12)	0.675
Glucose (mean (SD))		139.27<br>(57.28)	154.86<br>(71.71)	190.21<br>(109.26)	<0.001	168.10<br>(97.76)	155.88<br>(74.71)	157.69<br>(87.71)	0.783
SAPS II (mean (SD))		37.73<br>(11.98)	40.75<br>(13.19)	48.58<br>(14.56)	<0.001	42.66<br>(14.34)	41.63<br>(13.54)	42.78<br>(14.62)	0.741
GCS (mean (SD))		11.20<br>(3.93)	10.12<br>(4.02)	9.16<br>(4.25)	<0.001	10.27<br>(4.32)	10.18<br>(4.05)	9.96<br>(4.08)	0.836

Statistical analysis was performed using R 4.2.1, and a significance level of *P* < 0.05 was considered statistically significant.

## Results

### Baseline demographic and clinical characteristics

Table 2 displays the baseline characteristics of three groups categorized by LAR tertiles: tertile 1 (T1: LAR<0.411), tertile 2 (T2: 0.411 ≤ LAR<0.715), and tertile 3 (T3: LAR ≥ 0.715). The study included a total of 894 patients. Those with higher LAR exhibited increased RR, WBC, PT, BUN, Cr, sodium, glucose, and SAPS II score, along with longer lengths of ICU and hospital stays, but lower SBP and GCS scores. They were also more likely to have concurrent respiratory failure, sepsis, require mechanical ventilation, and were less likely to receive antiplatelet therapy. The overall ICU, hospital, 30-day, and 90-day mortality rates were 15.0% (134), 22.3% (199), 28.2% (252), and 36.1% (323), respectively. Participants with elevated LAR showed significantly higher rates of ICU, hospital, 30-day, and 90-day mortality (all *P *< 0.001).

**Table 2:** Baseline characteristics grouped by LAR.

	*N=894*	T1	T2	T3	p-value
		*N=298*	*N=299*	*N=297*	
Age (year)	73.6 [62.0; 82.5]	73.8 [62.9; 82.5]	72.4 [61.3; 81.7]	74.0 [62.1; 83.0]	0.568
Gender (n, %)					0.492
Female	407 (45.5)	138 (46.3)	128 (42.8)	141 (47.5)	
Male	487 (54.5)	160 (53.7)	171 (57.2)	156 (52.5)	
Hypertension (n, %)	471 (52.7)	157 (52.7)	154 (51.5)	160 (53.9)	0.846
Diabetes (n, %)	364 (40.7)	111 (37.2)	120 (40.1)	133 (44.8)	0.169
Heart failure (n, %)	336 (37.6)	112 (37.6)	117 (39.1)	107 (36.0)	0.736
Respiratory failure (n, %)	422 (47.2)	113 (37.9)	149 (49.8)	160 (53.9)	<0.001
Renal disease (n, %)	36 (4.03)	15 (5.03)	11 (3.68)	10 (3.37)	0.546
Sepsis (n, %)	259 (29.0)	38 (12.8)	71 (23.7)	150 (50.5)	<0.001
Shock (n, %)	285 (31.9)	33 (11.1)	85 (28.4)	167 (56.2)	<0.001
SBP (mmHg)	124 [106; 142]	127 [112; 147]	124 [107; 142]	120 [102; 138]	<0.001
DBP (mmHg)	68.0 [56.2; 81.0]	65.0 [55.2; 81.0]	71.0 [60.0; 83.0]	66.0 [55.0; 78.5]	0.005
RR (beats/min)	19.0 [16.0; 23.0]	17.0 [14.0; 21.0]	19.0 [16.0; 23.0]	20.0 [17.0; 24.0]	<0.001
WBC (×10^9^)	12.1 [8.20; 16.3]	10.1 [7.23; 13.6]	12.1 [8.60; 15.9]	14.1 [9.45; 20.9]	<0.001
HGB (g/dL)	10.6 [9.00; 12.3]	10.5 [8.90; 12.2]	10.9 [9.10; 12.6]	10.6 [9.10; 12.3]	0.185
PLT (×10^9^)	185 [131; 253]	182 [143; 240]	196 [134; 260]	175 [115; 263]	0.221
PT (s)	14.3 [12.6; 17.5]	13.3 [12.1; 15.6]	14.1 [12.4; 16.6]	15.8 [13.6; 21.0]	<0.001
BUN (mg/dL)	23.0 [15.0; 39.0]	20.0 [14.0; 30.8]	22.0 [15.0; 38.5]	28.0 [20.0; 48.5]	<0.001
Cr (mg/dL)	1.10 [0.80; 1.80]	1.00 [0.70; 1.50]	1.10 [0.80; 1.60]	1.40 [1.00; 2.10]	<0.001
Sodium (mmol/L)	139 [136; 142]	139 [136; 142]	139 [136; 142]	140 [136; 143]	0.005
Glucose (mg/dL)	137 [108; 184]	125 [103;1 57]	134 [108; 175]	152 [120; 236]	<0.001
Lactate/albumin	0.54 [0.36; 0.87]	0.31 [0.26; 0.36]	0.55 [0.47; 0.62]	1.10 [0.88; 1.57]	<0.001
Antiplatelet therapy (n, %)	616 (68.9)	233 (78.2)	212 (70.9)	171 (57.6)	<0.001
Mechanical ventilation (n, %)	523 (58.5%)	155 (52.0%)	172 (57.5%)	196 (66.0%)	0.002
SAPS II	41.0 [33.0; 51.0]	37.0 [30.0; 45.0]	39.0 [31.0; 49.0]	48.0 [38.0; 59.0]	<0.001
GCS	11.0 [7.0; 14.0]	13.0 [9.0; 14.0]	11.0 [7.0; 14.0]	10.0 [6.0; 13.0]	<0.001
Events					
Los ICU (days)	3.81 [2.05; 7.82]	2.95 [1.83; 6.15]	3.86 [2.24; 7.74]	4.56 [2.52; 8.69]	<0.001
Los hospital (days)	9.88 [5.69; 17.9]	8.22 [5.42; 14.6]	10.4 [5.88; 19.1]	11.7 [5.56; 19.8]	0.009
ICU mortality (n, %)	134 (15.0)	33 (11.1)	31 (10.4)	70 (23.6)	<0.001
Hospital mortality (n, %)	199 (22.3)	43 (14.4)	55 (18.4)	101 (34.0)	<0.001
Death_30d (n, %)	252 (28.2)	51 (17.1)	80 (26.8)	80 (26.8)	<0.001
Death_90d (n, %)	323 (36.1)	65 (21.8)	107 (35.8)	151 (50.8)	<0.001

### Association between LAR and all-cause mortality in critically ill patients with ischemic stroke

**Figure 2:**
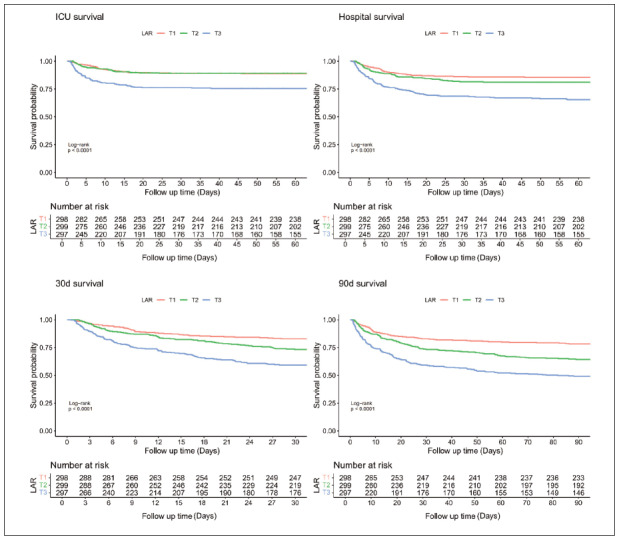
Kaplan–Meier curves of the ICU, Hospital, 30- and 90-day all-cause mortality by LAR tertiles.

Kaplan-Meier analysis was used to assess cumulative survival at different LAR levels, showing ICU, hospital, and 30- and 90-day survival curves for IS patients stratified by LAR tertiles (all *p* < 0.001; Figure 2). Higher LAR was associated with increased ICU mortality, with similar trends observed in hospital and 30- and 90-day survival curves.

**Figure 3:**
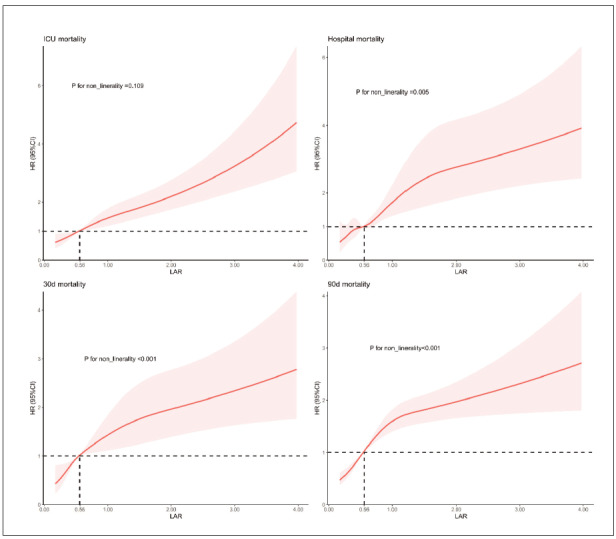
Restricted cubic spline curve illustrating the relationship between LAR and ICU, hospital, 30- and 90-day all-cause mortality. The vertical dotted lines represent LAR 0.56, selected as the reference level.

COX proportional-hazards regression was employed to analyze the association between LAR and ICU, hospital, and 30- and 90-day all-cause mortality, before and after adjusting for confounding factors. As shown in Table 3, when LAR was treated as a continuous variable, it was a significant risk factor for ICU, hospital, and 30- and 90-day mortality in unadjusted model I, as well as in model II (adjusted for age and gender), and model III (adjusted for age, gender, hypertension, diabetes, heart failure, respiratory failure, renal disease, sepsis, heart rate, SBP, DBP, RR, WBC, HGB, PLT, PT, BUN, Cr, sodium, glucose, SAPS II, and GCS). When LAR was treated as a categorical variable, using the low LAR tertile as reference, the high LAR tertile was associated with increased risk of ICU (HR: 2.43, 95% CI: 1.61–3.67, *P*<0.001), hospital (HR: 2.77, 95% CI: 1.94–3.95, *P*<0.001), 30-day (HR: 2.82, 95% CI: 2.03–3.91, *P*<0.001), and 90-day (HR: 2.91, 95% CI: 2.17–3.89, *P*<0.001) all-cause mortality in model I. The middle LAR tertile was associated with increased risk for 30-day (HR: 1.63, 95% CI: 1.15–2.31, *P*=0.007) and 90-day (HR: 1.75, 95% CI: 1.29–2.39, *P*<0.001) all-cause mortality. Similar results were observed in model II. In model III, the high LAR tertile was also associated with increased risk of 30-day (HR: 1.64, 95% CI: 1.10–2.43,* P*=0.013), and 90-day (HR: 1.74, 95% CI: 1.23–2.46, *P*=0.002) all-cause mortality compared to the low LAR tertile. Restricted cubic spline (RCS) analysis revealed a linear increase in ICU mortality risk with increasing LAR (*P* for non-linearity = 0.109), and a non-linear increase in hospital, 30-day, and 90-day mortality risks with increasing LAR (*P* for non-linearity = 0.005, *P* for non-linearity <0.001, and *P* for non-linearity <0.001, respectively; Figure 3).

**Table 3:** Association between LAR and the ICU, hospital, 30- and 90-day all-cause mortality in critically ill patients with IS. Model I: unadjusted; Model II: adjusted for age and gender; Model III: adjusted for age, gender, hypertension, diabetes, heart failure, respiratory failure, renal disease, sepsis, shock, antiplatelet therapy, mechanical ventilation, SBP, DBP, RR, WBC, HGB, PLT, PT, BUN, Cr, sodium, glucose, SAPS II, and GCS; LAR: T1 (LAR<0.411), T2 (0.411 ≤ LAR<0.715), and T3 (LAR ≥ 0.715)

Categories	Model I			Model II			Model III		
	HR (95%CI)	*P*-value	P for<br>trend	HR (95%CI)	*P*-value	P for<br>trend	HR (95%CI)	*P*-value	P for<br>trend
ICU mortality									
Continuous variable	1.59 (1.41,1.79)	<0.001		1.59 (1.41,1.80)	<0.001		1.28 (1.08,1.52)	0.004	
LAR			<0.001			<0.001			<0.001
T1(N=298)	Ref			Ref			Ref		
T2(N=299)	0.96 (0.59,1.57)	0.878		0.97 (0.59,1.58)	0.899		0.57 (0.34,0.96)	0.036	
T3(N=297)	2.43 (1.61,3.67)	<0.001		2.43 (1.61,3.68)	<0.001		1.16 (0.69,1.94)	0.570	
Hospital mortality									
Continuous variable	1.51 (1.36,1.68)	<0.001		1.54 (1.37,1.71)	<0.001		1.25 (1.07,1.45)	0.005	
LAR			<0.001			<0.001			<0.001
T1 (N=298)	Ref			Ref			Ref		
T2 (N=299)	1.33 (0.89,1.98)	0.166		1.35 (0.91,2.01)	0.14		0.85 (0.56,1.30)	0.458	
T3 (N=297)	2.77 (1.94,3.95)	<0.001		2.80 (1.96,4.00)	<0.001		1.43 (0.93,2.20)	0.100	
30d mortality									
Continuous variable	1.44 (1.29,1.60)	<0.001		1.47 (1.32,1.64)	<0.001		1.23 (1.07,1.43)	0.005	
LAR			<0.001			<0.001			<0.001
T1 (N=298)	Ref			Ref			Ref		
T2 (N=299)	1.63 (1.15,2.31)	0.007		1.67 (1.17,2.37)	0.004		1.07 (0.74,1.56)	0.705	
T3 (N=297)	2.82 (2.03,3.91)	<0.001		2.87 (2.07,3.98)	<0.001		1.64 (1.10,2.43)	0.013	
90d mortality									
Continuous variable	1.44 (1.30,1.58)	<0.001		1.47 (1.34,1.62)	<0.001		1.23 (1.08,1.40)	0.002	
LAR			<0.001			<0.001			<0.001
T1 (N=298)	Ref			Ref			Ref		
T2 (N=299)	1.75 (1.29,2.39)	<0.001		1.81 (1.33,2.46)	<0.001		1.22 (0.88,1.68)	0.236	
T3 (N=297)	2.91 (2.17,3.89)	<0.001		2.97 (2.22,3.98)	<0.001		1.74 (1.23,2.46)	0.002	

### Subgroup analyses

We conducted further risk stratification analysis of LAR for outcome measures in various subgroups of the study population, including age, gender, hypertension, diabetes, heart failure, respiratory failure, sepsis, shock, antiplatelet therapy, mechanical ventilation, SAPS II, and GCS. Our results indicated that age, gender, hypertension, diabetes, heart failure, sepsis, shock, antiplatelet therapy, mechanical ventilation, and SAPS II did not significantly modify the correlation between LAR and ICU all-cause mortality (*P* > 0.05 for all groups). However, significant interactions were observed between LAR and ICU (Table 4), hospital (Table 5), 30-day (Table 6), 90-day (Table 7) all-cause mortality in the subgroup with respiratory failure (*P* for interaction <0.001). In patients with varying GCS scores, we observed a significant interaction between LAR and 30-day mortality (*P* for interaction = 0.028; Table 6). Among patients with GCS >11, the T2 (HR: 2.68, 95% CI: 1.16–6.20) and T3 (HR: 1.96, 95% CI: 1.28–3.00) LAR tertiles were positively correlated with 30-day mortality. In patients with GCS ≤11, this correlation was observed only for the T3 tertile (HR: 1.33, 95% CI: 1.08–1.64). These results were consistent across different stratifications, indicating the stability and reliability of our study results.

**Table 4:** Subgroup analysis of the correlation between LAR and ICU all-cause mortality in critically ill patients with IS.

Variable Overall	N	LAR			*P* for interaction
	894	T1	T2	T3	
Age					0.702
>74	432	1.0 (ref)	0.74(0.34,1.61)	1.59(1.14,2.21)	
<=74	462	1.0 (ref)	0.75(0.36,1.56)	1.28(0.91,1.81)	
Gender					0.349
female	407	1.0 (ref)	0.45(0.20,1.00)	1.30(0.94,1.80)	
male	487	1.0 (ref)	1.17(0.52,2.38)	1.70(1.20,2.43)	
Hypertension					0.325
No	423	1.0 (ref)	0.34(0.13,0.88)	1.46(1.01,2.11)	
Yes	471	1.0 (ref)	1.03(0.53,2.00)	1.41(1.02,1.95)	
Diabetes					0.650
No	530	1.0 (ref)	0.73(0.38,1.42)	1.43(1.07,1.93)	
Yes	364	1.0 (ref)	1.12(0.45,2.80)	1.71(1.12,2.60)	
Heart failure					0.632
No	558	1.0 (ref)	0.70(0.38,1.29)	1.31(0.99,1.73)	
Yes	336	1.0 (ref)	0.89(0.32,2.42)	1.77(1.14,2.75)	
Respiratory failure					<0.001
No	472	1.0 (ref)	0.68(0.23,2.06)	2.00(1.33,3.00)	
Yes	422	1.0 (ref)	0.74(0.40,1.34)	1.16(0.87,1.56)	
Sepsis					0.164
No	635	1.0 (ref)	0.71(0.39,1.29)	1.53(1.16,2.02)	
Yes	259	1.0 (ref)	0.94(0.28,3.14)	1.56(0.90,2.70)	
Shock					0.373
No	609	1.0 (ref)	0.69(0.37,1.29)	1.36(1.00,1.83)	
Yes	285	1.0 (ref)	0.80(0.27,2.35)	1.38(0.87,2.17)	
Antiplatelet therapy					0.273
No	278	1.0 (ref)	0.97(0.46,2.05)	1.34(0.95,1.90)	
Yes	616	1.0 (ref)	0.53(0.24,1.17)	1.37(0.98,1.93)	
Mechanical ventilation					0.623
No	371	1.0 (ref)	0.26(0.05,1.25)	1.12(0.62,2.04)	
Yes	523	1.0 (ref)	0.82(0.46,1.47)	1.43(1.10,1.87)	
SAPS II					0.346
>41	419	1.0 (ref)	0.57(0.27,1.22)	1.41(1.05,1.89)	
<=41	475	1.0 (ref)	1.07(0.50,2.31)	1.24(0.80,1.92)	
GCS					0.074
>11	424	1.0 (ref)	0.32(0.03,3.28)	2.14(1.05,4.35)	
<=11	470	1.0 (ref)	0.62(0.35,1.08)	1.19(0.93,1.54)	

**Table 5:** Subgroup analysis of the correlation between LAR and hospital all-cause mortality in critically ill patients with IS.

Variable Overall	N	LAR			P for interaction
	894	T1	T2	T3	
Age					0.327
>74	432	1.0 (ref)	1.02 (0.57, 1.82)	1.47 (1.12, 1.94)	
<=74	462	1.0 (ref)	1.10 (0.58, 2.07)	1.67 (1.23, 2.26)	
Gender					0.390
female	407	1.0 (ref)	0.70 (0.37, 1.34)	1.49 (1.13, 1.96)	
male	487	1.0 (ref)	1.53 (0.84, 2.79)	1.72 (1.27, 2.33)	
Hypertension					0.403
No	423	1.0 (ref)	1.03 (0.51, 2.09)	1.73 (1.26, 2.39)	
Yes	471	1.0 (ref)	1.43 (1.09, 1.88)	1.43 (1.09, 1.88)	
Diabetes					0.439
No	530	1.0 (ref)	0.90 (0.53, 1.54)	1.45 (1.13, 1.86)	
Yes	364	1.0 (ref)	1.81 (0.85, 3.89)	2.02 (1.40, 2.91)	
Heart failure					0.787
No	558	1.0 (ref)	1.06 (0.64, 1.77)	1.47 (1.15, 1.88)	
Yes	336	1.0 (ref)	1.10 (0.51, 2.39)	1.67 (1.16, 2.39)	
Respiratory failure					<0.001
No	472	1.0 (ref)	1.18 (0.51, 2.70)	2.24 (1.58, 3.17)	
Yes	422	1.0 (ref)	0.96 (0.59, 1.57)	1.22 (0.95, 1.57)	
Sepsis					0.146
No	635	1.0 (ref)	1.01 (0.62, 1.64)	1.56 (1.22, 1.99)	
Yes	259	1.0 (ref)	1.33 (0.53, 3.32)	1.56 (1.03, 2.38)	
Shock					0.183
No	609	1.0 (ref)	1.08 (0.66, 1.77)	1.45 (1.12, 1.88)	
Yes	285	1.0 (ref)	0.97 (0.41, 2.27)	1.37 (0.94, 1.98)	
Antiplatelet therapy					0.817
No	278	1.0 (ref)	1.12 (0.59,2.14)	1.50 (1.11, 2.04)	
Yes	616	1.0 (ref)	0.95(0.53,1.70)	1.40 (1.05, 1.88)	
Mechanical ventilation					0.577
No	371	1.0 (ref)	1.15 (0.48, 2.76)	1.66 (1.10, 2.50)	
Yes	523	1.0 (ref)	0.99 (0.60, 1.64)	1.46 (1.15, 1.85)	
SAPS II					0.070
>41	419	1.0 (ref)	0.64 (0.34, 1.18)	1.45 (1.13, 1.86)	
<=41	475	1.0 (ref)	1.79 (0.96, 3.32)	1.50 (1.04, 2.15)	
GCS					0.334
>11	424	1.0 (ref)	1.15 (0.41, 3.19)	1.82 (1.14, 2.91)	
<=11	470	1.0 (ref)	0.86 (0.53, 1.39)	1.35 (1.08, 1.69)	

**Table 6:** Subgroup analysis of the correlation between LAR and 30-day all-cause mortality in critically ill patients with IS.

Variable Overall	N	LAR			*P* for interaction
	894	T1	T2	T3	
Age					0.541
>74	432	1.0 (ref)	1.28 (0.77, 2.11)	1.55 (1.21, 1.99)	
<=74	462	1.0 (ref)	1.40 (0.80, 2.45)	1.56 (1.17, 2.08)	
Gender					0.627
female	407	1.0 (ref)	0.98 (0.56, 1.71)	1.55 (1.20, 2.01)	
male	487	1.0 (ref)	1.68 (1.00, 2.85)	1.65 (1.25, 2.17)	
Hypertension					0.306
No	423	1.0 (ref)	1.49 (0.80, 2.75)	1.85 (1.36, 2.51)	
Yes	471	1.0 (ref)	1.20 (0.74, 1.94)	1.46 (1.14, 1.87)	
Diabetes					0.416
No	530	1.0 (ref)	1.13 (0.71, 1.81)	1.52 (1.21, 1.91)	
Yes	364	1.0 (ref)	2.33 (1.19, 4.54)	1.87 (1.34, 2.60)	
Heart failure					0.298
No	558	1.0 (ref)	1.13 (0.72, 1.77)	1.42 (1.13, 1.78)	
Yes	336	1.0 (ref)	1.88 (0.96, 3.66)	1.87 (1.34 ,2.61)	
Respiratory failure					<0.001
No	472	1.0 (ref)	1.05 (0.66, 1.68)	2.16 (1.62, 2.90)	
Yes	422	1.0 (ref)	1.05 (0.66, 1.68)	1.22 (0.95, 1.55)	
Sepsis					0.291
No	635	1.0 (ref)	1.23 (0.82, 1.87)	1.55 (1.24, 1.94)	
Yes	259	1.0 (ref)	1.89 (0.76, 4.71)	1.74 (1.14, 2.67)	
Shock					0.383
No	609	1.0 (ref)	1.34 (0.88, 2.04)	1.56 (1.25, 1.96)	
Yes	285	1.0 (ref)	1.28 (0.55, 2.99)	1.47 (1.00, 2.17)	
Antiplatelet therapy					0.939
No	278	1.0 (ref)	1.26 (0.70, 2.25)	1.49 (1.12, 1.97)	
Yes	616	1.0 (ref)	1.37 (0.84, 2.25)	1.44 (1.11, 1.88)	
Mechanical ventilation					0.365
No	371	1.0 (ref)	1.88 (0.99, 3.56)	1.80 (1.29, 2.50)	
Yes	523	1.0 (ref)	1.08 (0.66, 1.74)	1.41 (1.12, 1.78)	
Saps II					0.455
>41	419	1.0 (ref)	0.98 (0.59, 1.64)	1.43 (1.13, 1.80)	
<=41	475	1.0 (ref)	1.75 (1.00, 3.06)	1.58 (1.14, 2.17)	
GCS					0.028
>11	424	1.0 (ref)	2.68 (1.16, 6.20)	1.96 (1.28, 3.00)	
<=11	470	1.0 (ref)	0.91 (0.59, 1.40)	1.33 (1.08, 1.64)	

**Table 7:** Subgroup analysis of the correlation between LAR and 90-day all-cause mortality in critically ill patients with IS.

Variable Overall	N	LAR			*P* for<br>interaction
	894	T1	T2	T3	
Age					0.600
>74	432	1.0 (ref)	1.33 (0.86, 2.06)	1.53 (1.23, 1.91)	
<=74	462	1.0 (ref)	1.63 (0.98, 2.70)	1.68 (1.29, 2.17)	
Gender					0.937
female	407	1.0 (ref)	1.29 (0.80, 2.08)	1.59 (1.25, 2.00)	
male	487	1.0 (ref)	1.56 (0.99, 2.47)	1.66 (1.31, 2.11)	
Hypertension					0.261
No	423	1.0 (ref)	1.53 (0.89, 2.65)	1.87 (1.42, 2.47)	
Yes	471	1.0 (ref)	1.30 (0.86, 1.98)	1.44 (1.15, 1.79)	
Diabetes					0.851
No	530	1.0 (ref)	1.40 (0.92, 2.11)	1.97 (1.12, 3.45)	
Yes	364	1.0 (ref)	1.97 (1.12, 3.45)	1.83 (1.39, 2.43)	
Heart failure					0.349
No	558	1.0 (ref)	1.28 (0.85, 1.91)	1.42 (1.16, 1.75)	
Yes	336	1.0 (ref)	1.78 (1.01, 3.16)	1.88 (1.41, 2.52)	
Respiratory failure					<0.001
No	472	1.0 (ref)	1.67 (0.98, 2.86)	2.07 (1.61. 2.66)	
Yes	422	1.0 (ref)	1.30 (0.86, 1.96)	1.26 (1.01, 1.56)	
Sepsis					0.494
No	635	1.0 (ref)	1.41 (0.98, 2.03)	1.50 (1.22, 1.85)	
Yes	259	1.0 (ref)	1.59 (0.74, 3.41)	1.61 (1.14, 2.27)	
Shock					0.301
No	609	1.0 (ref)	1.43 (0.99, 2.06)	1.54 (1.26, 1.90)	
Yes	285	1.0 (ref)	1.29 (0.62, 2.69)	1.37 (0.98, 1.91)	
Antiplatelet therapy					0.885
No	278	1.0 (ref)	1.45 (0.84, 2.48)	1.55 (1.18, 2.03)	
Yes	616	1.0 (ref)	1.38 (0.91, 2.10)	1.48 (1.18, 1.85)	
Mechanical ventilation					0.601
No	371	1.0 (ref)	1.63 (0.96, 2.77)	1.90 (1.45, 2.49)	
Yes	523	1.0 (ref)	1.30 (0.85, 2.00)	1.42 (1.14, 1.76)	
Saps II					0.798
>41	419	1.0 (ref)	1.19 (0.75, 1.89)	1.46 (1.17, 1.82)	
<=41	475	1.0 (ref)	1.64 (1.02, 2.64)	1.62 (1.24, 2.12)	
GCS					0.060
>11	424	1.0 (ref)	2.54 (1.36, 4.73)	1.96 (1.41, 2.73)	
<=11	470	1.0 (ref)	1.00 (0.68, 1.48)	1.34 (1.11, 1.63)	

## Discussion

In this study, we investigated the relationship between the LAR and short- and long-term all-cause mortality in critically ill IS patients using data from a United States (US) cohort. We observed that LAR was significantly associated with ICU, Hospital, 30-day, and 90-day all-cause mortality. Specifically, higher LAR values were specifically associated with increased ICU, hospital, 30-day, and 90-day mortality risks in critically ill IS patients. This association with 30- and 90-day all-cause mortality remained significant after adjusting for confounding factors. Further analysis using RCS showed that while LAR had a linear association with ICU all-cause mortality risk, its association with hospital, 30-day, and 90-day allcause mortality was non-linear. Subgroup analysis confirmed the stability of the correlation between LAR and all-cause mortality. Therefore, LAR emerged as a robust independent predictor of short- and longterm all-cause mortality in critically ill IS patients, suggesting its potential as a valuable tool for clinical decision-making.

Lactate, a byproduct of anaerobic respiration, is produced in large quantities under hypoxic conditions [25]. It plays a crucial role in regulating various biological and pathological processes and is produced as a waste product of glucose metabolism, a process promoted by hypoxia, inflammation, viral infections, and tumors [26]. Lactate is produced by the reduction of pyruvate via the enzyme lactate dehydrogenase. In critically ill conditions with hypoperfusion and hypoxia, pyruvate accumulates rapidly, leading to a shift in metabolism towards lactate production. This results in a significant increase in intracellular lactate levels, which is then excreted into the bloodstream [7]. Historically, elevated lactate levels have been associated with poor outcomes and increased mortality [27], making blood lactate a strong predictor of mortality in critically ill patients [28] [29]. During pathophysiological processes, lactate not only serves as an energy substrate in the brain but also plays a role in maintaining long-term memory formation and cognitive function. It can also act as a signaling molecule to reduce excitatory injury, suggesting its involvement in overall brain metabolism and functional regulation [30]. In ischemia/reperfusion injury, lactate- induced release of inflammatory cytokines like TNF-α, IL-6, and IL-1 further exacerbates neuronal damage in acute stroke [31]. Clinically, serum lactate measurement is crucial in guiding the treatment of patients with ischemic injury, particularly in managing tissue hypoxia and ischemia-reperfusion injury caused by cerebral ischemia and inadequate blood flow in myocardial infarction patients [32] [33] [34] [35]. However, the regulation of serum lactate levels is complex. Most of the lactate released into circulation is metabolized by the liver (60%) and kidneys (30%) [36], so patients with liver or kidney disease may have abnormal lactate metabolism. Additionally, various conditions such as cardiac arrest, trauma, excessive muscle activity, seizures, regional ischemia, liver dysfunction, diabetic ketoacidosis, metformin use, burns, smoke inhalation, and thiamine deficiency can lead to high lactate levels [36] [37]. Moreover, the increase in lactate concentrations may be due to factors other than cellular hypoxia, so the decrease in blood lactate concentrations is more than a result of improved cellular oxygen availability [6]. For example, beta-adrenergic stimulation may increase lactate production [38], the infusion of lactate-containing intravenous solutions also potentially complicate the interpretation of blood lactate concentrations [39], even blood alcohol levels affect the rate of decline in lactate levels [40]. Thus, the evaluation of blood lactate levels is challenging, and relying solely on lactate to predict patient prognosis may be unreliable.

Albumin, the most abundant plasma protein, constitutes approximately 50–60% of total serum plasma proteins [41] [42]. Synthesized initially as preproalbumin by hepatocytes, it is subsequently cleaved to form proalbumin, which is then converted into albumin. While the majority of albumin is water-soluble and secreted into circulation, a small amount remains in the liver [41] [43]. Albumin’s functions are diverse and include binding to endogenous and exogenous substances, antioxidant activity, metabolic functions, anticoagulant effects, regulation of inflammatory cells, acid-base balance, and actions crucial for fluid transport, oncotic pressure, and microvascular integrity [44] [45]. The levels of albumin in plasma and urine reflect both liver protein synthesis and vascular endothelial function. Traditionally, plasma or serum albumin levels have been used as a classicmarker of nutritional status. Recently, low albumin levels have been increasingly recognized as a risk factor and predictor of morbidity and mortality [46]. Hypoalbuminemia is associated with various disease processes, including those leading to systemic inflammatory response syndrome, gastrointestinal disorders, hepatic disorders, and glomerular diseases [47]. While serum albumin is a general marker of disease severity, hypoalbuminemia is particularly associated with a poor prognosis. However, as albumin is an acute phase protein, its levels in critically ill patients fluctuate with illness severity [48]. Since albumin is synthesized in the liver and cleared through catabolic processes, as well as the gastrointestinal and renal systems [49], its levels are influenced by hepatic and renal function, and gastrointestinal function. This reduces the reliability of using lactate alone to predict patient prognosis.

Considering that lactate and albumin are produced from different organs and affected by multiple mechanisms, to more accurately predict the prognosis of patients with IS, a ratio of blood lactate and serum albumin (LAR) can be used to reduce the influence of a single factor on the regulatory mechanism. In our analysis, LAR emerged as a potential independent predictor for 30- and 90-day all-cause mortality in patients with IS. Factor regression analysis established a significant correlation between LAR and short- and long-term mortality. Subgroup analyses showed that age, gender, hypertension, diabetes, heart failure, sepsis, shock, antiplatelet therapy, mechanical ventilation and SAPS II had no significant interactions with the correlation of LAR and all-cause mortality, however, significant interactions were observed between LAR and 30-day all-cause mortality in the GCS subgroups, which may reflect variations in ischemic stroke severity. Further studies are needed to confirm these findings.

Several limitations should be noted. First, this study was a retrospective, single-center study based on the MIMIC-IV database, leading to inevitable selection bias. Second, although the predictive role of LAR in critically ill IS patients was discussed, the relevant internal mechanisms were not elucidated. Third, this study investigated the relationship between LAR within 24h of admission and patient prognosis, without assessing the prognostic impact of dynamic changes in this ratio during hospitalization. Further studies are necessary to validate the relationship between LAR and IS. Despite these limitations, this study is meaningful as LAR could serve as an independent predictor for critically ill IS patients.

## Conclusions

In conclusion, the LAR shows a significant association with 30- and 90-day all-cause mortality in critically ill patients with IS. Thus, LAR has the potential to serve as a predictor for both short- and long-term mortality in these patients, offering clinicians a promising tool for decision-making.

## Dodatak

### Ethics statement

All the data presented in this study were extracted from a third-party anonymised publicly available database (MIMIC-IV). Informed consent was not required in this database study because of the nonidentifying and anonymous nature of the database.

### Data availability statement

The raw data supporting the conclusions of this article will be made available by the authors, without undue reservation.

### Funding

This research received no specific grant from any funding agency in the public, commercial or not-for-profit sectors.

### Author contributions

LK conceptualized the research aims, planned the research. SQ, JX, SY conducted data analysis,wrote the manuscript, and contributed equally to this study. HZ conducted data collection. YL, NL, SH modified the manuscript and studied review. All authors read and approved the final version of the manuscript.

Sisi Qin, Jijie Xiao and Shiqi Yuan contributed equally to this work.

### Conflict of interest statement

All the authors declare that they have no conflict of interest in this work.
